# A Panel of rSNPs Demonstrating Allelic Asymmetry in Both ChIP-seq and RNA-seq Data and the Search for Their Phenotypic Outcomes through Analysis of DEGs

**DOI:** 10.3390/ijms22147240

**Published:** 2021-07-06

**Authors:** Elena E. Korbolina, Leonid O. Bryzgalov, Diana Z. Ustrokhanova, Sergey N. Postovalov, Dmitry V. Poverin, Igor S. Damarov, Tatiana I. Merkulova

**Affiliations:** 1 The Federal Research Center Institute of Cytology and Genetics, The Siberian Branch of the Russian Academy of Science, 10 LavrentyevaProspekt, 630090 Novosibirsk, Russia; leonolr14@gmail.com (L.O.B.); damarovis@bionet.nsc.ru (I.S.D.); merktativ@gmail.com (T.I.M.); 2 VECTOR-BEST, PO BOX 492, 630117 Novosibirsk, Russia; 3 Department of Information Biology, The Novosibirsk State University, 1 Pirogovast, 630090 Novosibirsk, Russia; dianaa45rus@gmail.com; 4 Department of Theoretical and Applied Informatics, The Novosibirsk State Technical University, 630073 Novosibirsk, Russia; post@fpm.ami.nstu.ru (S.N.P.); poverin.2012@corp.nstu.ru (D.V.P.)

**Keywords:** allele-specific events, regulatory SNPs, Genotype-Tissue expression, eQTLs, enrichment analysis, protein-protein interaction networks, molecular phenotype

## Abstract

Currently, the detection of the allele asymmetry of gene expression from RNA-seq data or the transcription factor binding from ChIP-seq data is one of the approaches used to identify the functional genetic variants that can affect gene expression (regulatory SNPs or rSNPs). In this study, we searched for rSNPs using the data for human pulmonary arterial endothelial cells (PAECs) available from the Sequence Read Archive (SRA). Allele-asymmetric binding and expression events are analyzed in paired ChIP-seq data for H3K4me3 mark and RNA-seq data obtained for 19 individuals. Two statistical approaches, weighted z-scores and predicted probabilities, were used to improve the efficiency of finding rSNPs. In total, we identified 14,266 rSNPs associated with both allele-specific binding and expression. Among them, 645 rSNPs were associated with GWAS phenotypes; 4746 rSNPs were reported as eQTLs by GTEx, and 11,536 rSNPs were located in 374 candidate transcription factor binding motifs. Additionally, we searched for the rSNPs associated with gene expression using an SRA RNA-seq dataset for 281 clinically annotated human postmortem brain samples and detected eQTLs for 2505 rSNPs. Based on these results, we conducted Gene Ontology (GO), Disease Ontology (DO), and Kyoto Encyclopedia of Genes and Genomes (KEGG) pathway enrichment analyses and constructed the protein–protein interaction networks to represent the top-ranked biological processes with a possible contribution to the phenotypic outcome.

## 1. Introduction

Single nucleotide polymorphisms (SNPs) are the most common type of sequence variation. The number of SNPs so far contained in the NCBI dbSNP is over 150 million (dbSNP. Available online: https://www.ncbi.nlm.nih.gov/snp/ (accessed on 31 May 2021). Since it is highly likely that most SNPs lack any functionality [1], one of the major problems in human genetics is the identification of the functionally relevant variants from the multitude of available ones. The main and historically first approach to solving this problem on a genome-wide scale is GWAS, genome-wide association studies, making it possible to detect the association between genetic variations and traits. The advent of GWAS has allowed approximately 70,000 SNP-disease/trait associations to be identified in the last 15 years [2,3]. However, this approach fails to distinguish between causal polymorphisms and numerous marker SNPs detected according to linkage disequilibrium (LD). Moreover, GWAS technology is unable to give any information about the molecular mechanisms that determine the effect of these variants on the risks of diseases and, thus, makes it necessary to perform laborious follow-up studies for each selected individual variant [4,5,6]. This is especially important in the case of noncoding SNPs, which account for about 90% of the GWAS-associated genetic variants [7,8] and the functional interpretation of which is the most complex task. Note that the functional interpretation is necessary for both an increase in the prognostic value of polymorphisms and the possibility to design new methods for correcting the associated clinical outcomes.

To make molecular sense of GWAS, many recent studies are focused on functional analysis of the SNPs with a known GWAS disease/trait associations, including both (i) individual variants [9,10,11,12,13,14,15] and (ii) large SNP arrays, with the help of state-of-the-art approaches of functional genomics. These methods comprise various functional annotations, including transcription factor (TF) binding motifs, histone modifications, promoters, enhancers, chromatin accessibility landscapes, and three-dimensional chromatin interactions [16,17,18,19,20]; expression quantitative trait loci (eQTLs) mapping [21,22], and several other approaches, such as massively parallel reporter assay (MPRA) [23], SNPs-seq [24], and SNPs-SELEX [25].

Concurrently, alternative genome-wide approaches have beendeveloped with the primary focus being thedetermination of the functionality of genetic variants [26,27,28,29,30,31,32,33,34,35]. In these approaches, (i) the estimation of the effect of SNPs on TF binding using position weight matrix (PWM) models [26,30]; (ii) falling of SNPs into regulatory regions [31,36,37], and (iii) allele-specific expression (ASE) [32],and (iv) allele-specific binding (ASB) events in ChIP-seq, DNase-seq, and ATAC-seq data [26,27,28,29,33,34,35] are used.

In our earlier work, we searched for ASB events in K562, MCF-7, and HCT-116 human cell lines by analyzing the ENCODE ChIP-Seq data for epigenetic histone marks (H3K27ac, H3K4me1, H3K4me2, H3K4me3, and H3K27me3) and 456 different chromatin-associated proteins, mainly TFs [33]. The ASE events were also assessed using RNA-Seq and ChIA-PET data obtained using HCT-116, MCF-7, and K562 cells (ENCODE). This allowed 1633 rSNPs simultaneously associated with both types of allele-specific events to be identified. According to GWAS, 27 of them were associated with a risk of malignancy [33] and 14 with cognitive disorders [38]. However, both of these studies, as well as most others [27,28,29,35], used cancer cell lines with a high level of genomic instability (aneuploidy, gene amplification, generation of extrachromosomal elements, and numerical chromosomal defects) [39,40,41,42], which considerably complicates the search for allele-specific events in the genome-wide data sets thus obtained. Moreover, when cancer cell lines are used, rSNPs exhibit their functionality under conditions far from the biological context of normal tissue.

That is why we took the data of ChIP-seq and RNA-seq experiments with clinical material, biopsies—human pulmonary arterial endothelial cells, PAECs [43]—the use of which in the search for allelic asymmetry is free from this shortcoming. We have used a modified approach, which allows the overall genome to be analyzed rather than only certain selected regulatory regions as compared with the earlier used method [33] and provides a more reliable identification of the rSNPs associated with both types of allele-asymmetric events. In addition, we have attempted to relate the detected rSNPs to molecular phenotype by comparing them to the known eQTLs [32], assessing their ability to act as eQTLs in an independent transcriptome dataset [44], and constructing gene networks via analysis of DEGs (differentially expressed genes) by functional annotations.

## 2. Results

### 2.1. Workflow for rSNP Identification

#### 2.1.1. General Description

The NCBI datasets for biopsy material (PAECs) were analyzed here in several steps, largely using those fromour earlier study of cancer cell lines [33]. Thus, the most important differences in the search for rSNPs were (i) the use of NGS data for human tissue samples instead of cell lines and (ii) analysis of the whole genome. Earlier, we focused on the genomic regions containing two or more overlapping TF binding regions (OTFRs) [37]. Additionally, weighted *z*-scores (*z*-scores) and predicted probabilities were calculated to aid rSNP filtering.

**Figure 1:**
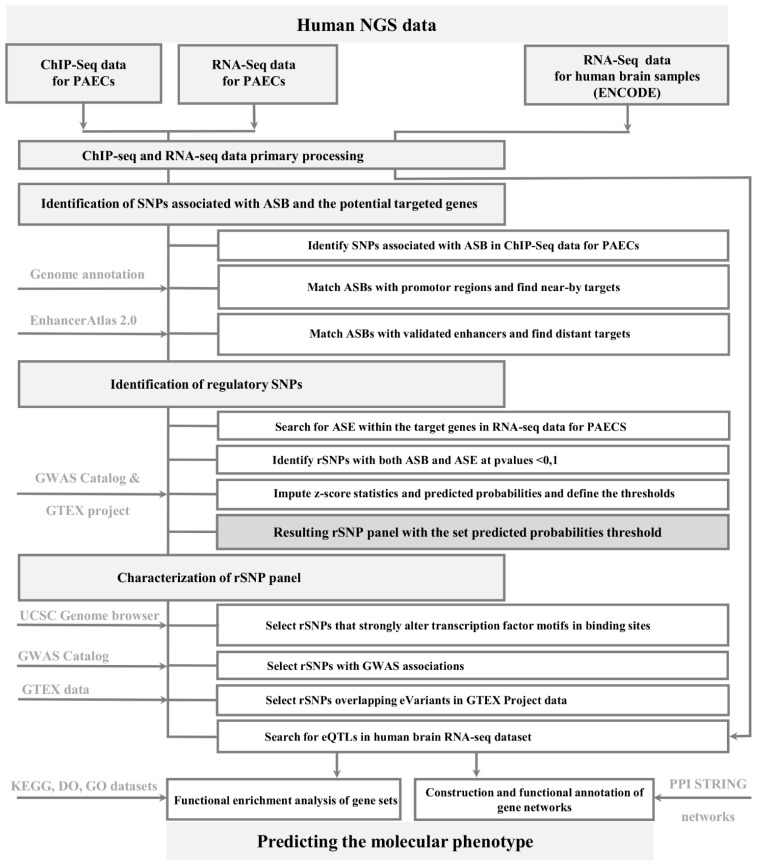
The stages of rSNP search assessing the likely molecular phenotypes and phenotypic outcomes.Arrows show the utilization of sequencing data or publicly available resources; NGS:next generation sequencing; PAECs:pulmonary arterial endothelial cells; SNP:single nucleotide polymorphism; rSNP:regulatory SNP; ASB:allele-specific binding bias; ASE:allele-specific expression bias; PPI;protein-protein interaction.

In general, the search algorithm processed two main inputs: the raw reads from ChIP-seq and RNA-seq experiments separately for each PAECs sample (Figure 1) to assess the regulatory SNPs associated with both ASB and ASE. It started with (1) preprocessing, namely, the raw reads were trimmed and filtered; (2) the high-quality reads were mapped to thehuman hg38 reference; and (3) personal alternative genomes for each sequencing dataset were constructed using identified heterozygous SNPs variants to realign the ChIP-seq or RNA-seq datasets to the appropriate alternative genomes to reduce the reference bias. Because each individual could have multiple ChIP-seq or RNA-seq datasets, the alignment was performed to the corresponding personal genome twice for each of 54 ChIP-seq and 88 RNA-seq experiments. As a result, the data were converted into tables, including the frequency of the reference and alternative alleles for each heterozygous position and coverage. (4) The ASB events were found from ChIP-seq profiling of H3K4me3, a signature of active promoters. (5) The heterozygous SNPs with ASB were searched for in gene promoter regions. The genes harboring ASB events in promoters were considered nearby targets. (6) ASB events were also searched for within the validated enhancer regions using EnhancerAtlas 2.0 tools. The genes harboring ASB events within enhancers were considered distant targets. (7) The algorithm processed human PAEC transcriptomic data to assess the ASE events in the target genes. (8) The SNPs associated with both ASB and ASE (*p*-value < 0.1 in binomial test in both cases) in total in 19 PAEC samples were designated as rSNPs. (9) Next, taking into account the errors, the summary *z*-score statistics and predicted probabilities were imputed to assess the likelihood that the rSNPs identified through a comprehensive analysis of independent ChIP-seq and RNA-seq experiments did have an impact on gene expression and trait. The cutoffs were determined based on GTExeQTL datasets and GWAS.

#### 2.1.2. Using GWAS and eQTLs to Define Cutoff Thresholds

Analysis of promoter regions allowed us to identify 12,993 rSNPs associated with allele specificity in both the distribution of histone modifications and transcriptome data. Analysis of enhancers increased the total to 20,321. Our rationale was thatthe imputed integral parameters (*z*-scores or predicted probabilities) should reflect the likelihood that a certain rSNP is a true functional variant with the effects on target gene expression and/or trait. If this is the case, the lower the z-score or, the higher the predicted probability value, the more the resulting rSNP set is enriched in GWAS phenotype associations and/or eQTLs (as the experimentally determined effects of SNP on gene expression).

**Figure 2:**
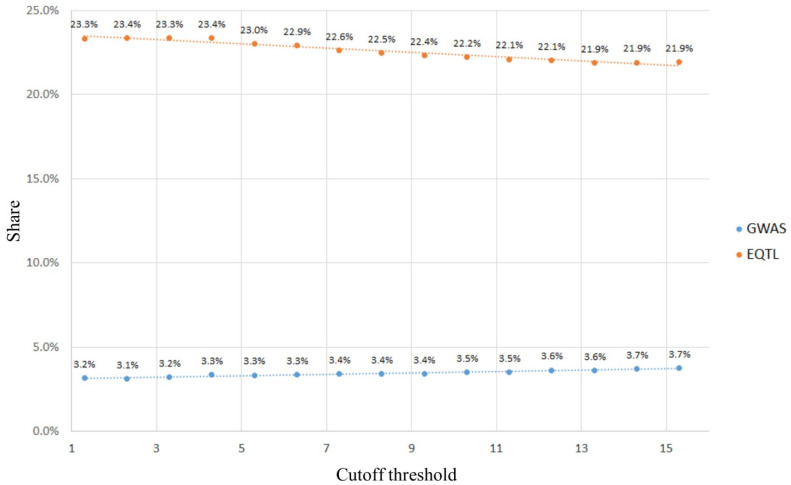
Enrichment of the resulting rSNP set in GWAS phenotype associations and/or eQTLs.The cutoff threshold was−log α, where α is the probability of the first type.

We calculated the weighted *z*-scores [45] since this parameter is an integral characteristic of statistical significance (*p*-values) for ASB and ASE. The enrichment is shown in Figure 2.

Analysis of the data shown in Figure 2 allows us to conclude that the share of eQTLs SNP slightly decreases with an increase in the cutoff threshold, while the share of GWAS SNPs slightly increases. Selecting the cutoff threshold of *z*-score = 5 × 10^−6^,taking into account the Bonferroni correction, we obtainedalist of 11,266 rSNPs, 2592 (23%) of which wereGTEX eQTLs and 375 (3.3%) wereannotated in GWAS.

#### 2.1.3. Setting Predicted Probabilities from Logistic Regression

A predicted probability is the probability of an event calculated from available data [46]. First, we used logistic regression to predict the probability of each rSNP to be a GTExeQTL using three independent parameters. Two parameters, |log_2_FC1| and |log_2_FC2|, showed the degree of asymmetry in ChIP-seq and RNA-seq, respectively (where FC is fold change). In other words, these parameters reflect the proportion of the number of filtered reads containing different alleles of a polymorphic position (rSNP). The difference between the between |log_2_FC1| and |log_2_FC2| was shown by the |log_2_FC1/FC2| parameter. An eQTL was a binary label. The results are consolidated in Table 1.

We additionally performed a logistic regression without using the |log_2_FC1/FC2| parameter. ANOVA showed a significant influence of |log_2_FC1/FC2| on the likelihood of an rSNP to be an eQTL (Chi = 138.29, *p* < 2.2 × 10^−16^), which means that the use of |log_2_FC1/FC2| considerably increases the strength of prediction. Note that this implies that if an rSNP is an eQTL, than the ratio between the degree of asymmetry value in ChIP-seq and RNA-seq for it is not random.

**Figure 3:**
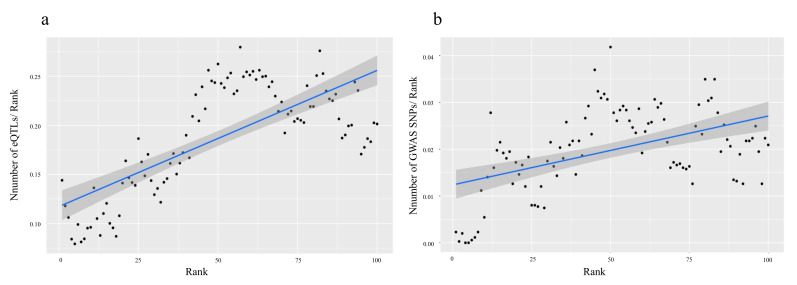
Q-Q plots showing a significant positive correlation of the number of (**a**) eQTLs and (**b**) GWAS SNPs in the resulting rSNP set with predicted probabilities. All rSNPs were divided into 100 equal parts or ranks (the abscissa); the ordinate shows the number of (**a**) GTExeQTLs or (**b**) GWAS associations mapped to all SNPs of a particular rank.

Correspondingly, the predicted probabilities for all rSNPs were calculated using three parameters (|log_2_FC1|, |log_2_FC2|, and |log_2_FC1/FC2|) and rank correlation was performed (Figure 3a). Correlation analysis showed a moderate to strong positive correlation between the predicted probabilities and the likelihood of rSNP being eQTL from GTEx collection. The rank correlation for GWAS was calculated using the same model (Figure 3b).

We observed the trend of an increase in the number of eQTLs for the rank up to the rank of 50. Thus, the corresponding minimum value of predicted probabilities of >0.1929408 was chosen as the cutoff threshold to select the final rSNP set, which included 14,266 rSNPs. Note that a similar trend was observable for the GWAS associations per rank up to the rank of 50; however, the correlation coefficient was lower, and the dispersion was higher.

Next, we analyzed the proportion of the SNPs associated with GWAS traits and GTExeQTL effects at four stages of our rSNP search.We found out that the proportion of the SNPs contained in public collections gradually increased from ‘identifying all heterozygous SNPs from the dataset’ to ‘using the cutoffs’ for both eQTLs and GWAS (Table 2). At the step of ‘rSNP identification’ when the positions associated with both types of asymmetric effects are considered, the eQTL enrichment at any *p*-value threshold almost doubled when comparing with ‘searching only ASBs’ or ‘searching only ASEs’ stages. Note that the enrichment further increased when ‘using the cutoffs’ for both z-score and prediction probabilities. According to GWAS data, a similar trend was also observed; however, all counts were noticeably lower at any step.

This confirms our preliminary conclusion that analysis of two types of allele-specific events and the use of a certain threshold of predicted probability allows for an increase in the efficiency of rSNP identification. Analysis of the data listed in Table 2 demonstrates that the use of *z*-score is less efficient as compared with the predicted probabilities. Correspondingly, we used only the predicted probabilities threshold for constructing the resulting panel of rSNPs.

### 2.2. Characterization of the Resulting rSNP Panel

**Figure 4:**
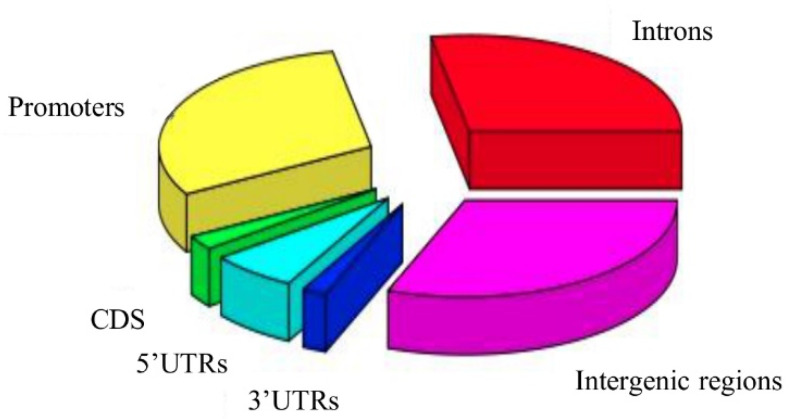
Distribution of 14,266 rSNPs across human genomic regions.

As is mentioned, the threshold was set at prediction probabilities of >0.1929408 beyond which 14,266 rSNPs entered the final rSNPs panel (Supplementary Table S1). These rSNPs are of different classes with respect to their location, namely, intronic (7202), located within untranslated regions: 5′ UTR (1713) or 3′ UTR (481), promoter regions (7818) or CDS (618), and intergenic regions (7969) (Figure 4). Our results on ASE suggest that the identified rSNPs may influence the expression of 7852 nearby genes from one to five per position, and 4981 rSNPs are located within the consensus human enhancers from the EnhancerAtlas 2.0 collection [47].

#### 2.2.1. Search for rSNPs within Known TF Binding Motifs

When a TF motif houses SNPs, its DNA binding site may be affected; correspondingly, it is assumed that the expression of the target genes is also changed. A motif analysis with the motifbreakeR showed that 11,536 rSNPsaltered the binding motifs of 374 different TFs. Additionally, we found that 205 rSNPs mapped to ChIP-seq peaks for the corresponding TFs according to ENCODE data, and 68 rSNPs from these strongly altered the binding motifs (Table S1).

#### 2.2.2. Overlapping with GWAS Variants

To estimate the potential associations between an rSNP and a trait, we first searched for our rSNPs in the GWAS Catalog. We found that 645 variants (4.5% of 14,266) contained in the GWAS Catalog. These variants were associated with various phenotypic GWAS traits, including schizophrenia; the risk of various cancers; Alzheimer’s disease; asthma or atopic dermatitis; inflammatory and autoimmune diseases; white matter microstructure; and cardiovascular diseases (Table S2).

In order to expand the number of GWAS associations in our rSNP set, we imputed the normalized Hamming distances (NHDs) (see Section 4) between rSNPs and 135,132 GWAS-derived SNP markers available at the moment to measure the pairwise linkage. As a result, 405 different linked SNP pairs were extracted for 293 GWAS-derived SNPs using the 1000 Genomes data with a weighted Hamming distance of ≤0.001. Among them, 57 variants with an NHD of zero paired with themselves.

#### 2.2.3. Finding rSNPs in GTExeQTLCollection

**Figure 5:**
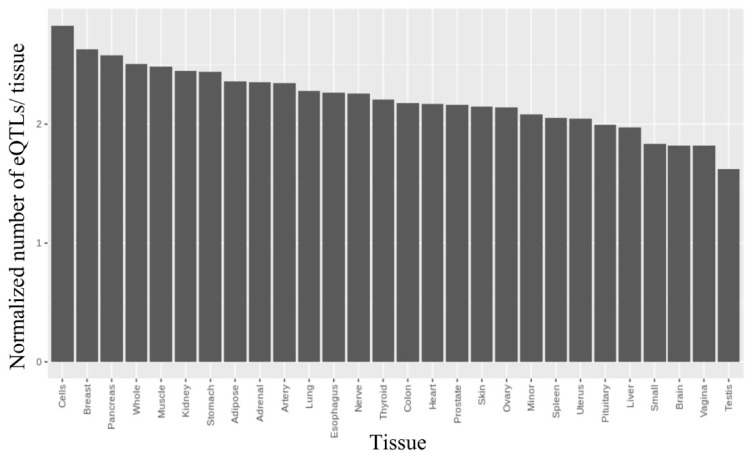
Normalized tissue distribution of the rSNPs mapped as GTExeQTLs.The ordinate shows the number of eQTLs (padj < 0.1) identified for all rSNPs in the tissue normalized on the number of all GTExeQTLs mapped in the tissue with padj < 0.1; the abscissa shows the tissue source with grouping by type.

Then, we integrated our final rSNP set with the eQTL mapping data from the GTEx Project. In total, we identified 4746 genome-wide significant eQTLs (unique SNP-gene pairs with a false discovery rate of <0.05) [32] in different tissues. The maximum number of eQTLs associated with an individual rSNP in all GTEx tissues was five. The results showed no pronounced enrichment in any tissue (Figure 5). However, note that the ‘artery’ tissue close to PAECs in its type was among the GTEx tissues in which we found the largest number of eQTLs (at least in the left part of the diagram).

### 2.3. Assessing eQTLs in Human Brain RNA-seq Dataset

To search for additional eQTLs, we analyzed a representative independent RNA-seq dataset from the study by Ramaker et al. [44]. However, since most of the identified rSNPs were located within noncoding genomic regions, we used the specific coding markers to predict the allelic combination for rSNP positions in each individual.

We hypothesized that the presence of a certain SNP in the genomic coding region in RNA-seq data could predict the presence of a linked rSNP in the same individual genome. To that end, we called for SNPs within the transcribed regions of known human genes based on 2504 individual genomes from the four 1000 Genomes Project super populations. The composite distance measure to determine the linked variants was an NHD of 0.1 (Section 4). As a result, 771,471 coding SNP markers were extracted for the identified rSNPs from the 1000 Genomes project.

Then, the patients were genotypedin silico according to each rSNP separately and were divided into ‘homozygous‘ and ‘heterozygous’ groups depending on the presence of specific rSNP alleles where possible. The following criteria were used: (i) an rSNP or a marker variant was found in no less than six individuals, and (ii) a minor rSNP allele was found in no less than three individuals. If the coverage of the minor allele was <10%, the genotype was considered homozygous, and if the coverage of the minor allele was ≥10%, heterozygous. The coverage of up to the top ten coding markers for a certain rSNP was combined with increasing the position coverage. Next, for each rSNP, differential gene expression between groups of different genotypes was analyzed. As a result, differently expressed genes (DEGs) were identified for 2505 rSNPs by DeSeq 2.0 with the Bonferroni *p*-value correction (padj < 0.1). A network enrichment analysis was performed for rSNP group with over a hundred DEGs.

To functionally interpret the resulting lists of DEGs, we further carried out functional enrichment analysis using theclusterProfiler R tool. In the issue, we accessed the functional annotations for 2246 rSNPs (Table S3), including 1398 in the KEGG pathway, 462 in GO, and 1237 in DO annotations. This made it possible to refill the list of potential rSNP annotations.

With the aim of further interpretation of the observed eQTL effects, we analyzed a set of DEGs independently for each rSNP using protein-protein interaction (PPI) network analysis. The subsets of the associated proteins for each rSNP were plotted as STRING networks using STRINGdb with functional enrichment analysis when possible.

**Figure 6:**
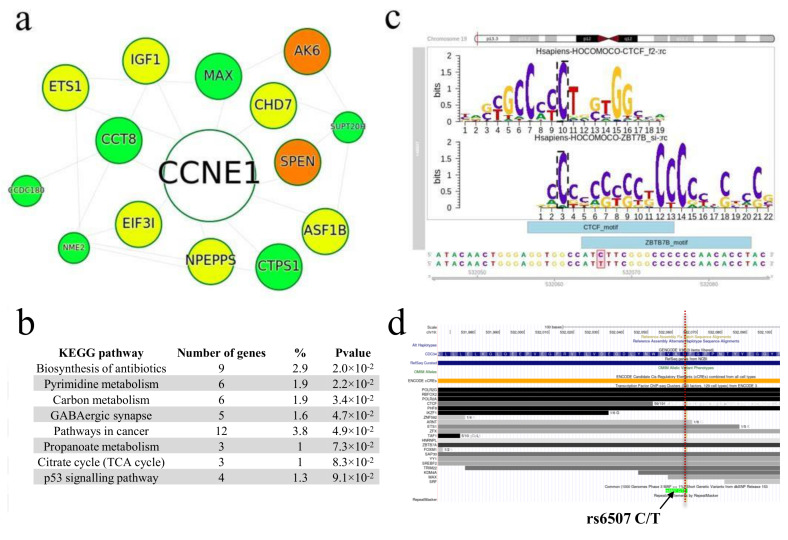
rs6507 (C>T) findings. (**a**) PPI subnetwork for rs6507 with the ‘root’ CCNE1 protein using R STRINGdb. Nodes are colored according to logFC value (green nodes: logFC < −0.5; orange nodes: logFC > 0.5; yellow nodes: |logFC| < 0.5) and node sizes are proportional to |logFC|. (**b**) The enriched KEGG functional terms for 337 corresponding DEGs are ranked according to the adjusted *p*-value and displayed in a tabular format. (**c**) Disruption of CTCF and ZBTB7B binding motifs by rs6507 (C>T). The red bar shows the chromosome location of rs6507. (**d**) The genome region surrounding rs6507 with visualized ChIP-Seq signal tracks for CTCF as given by ENCODE annotation (ICGC Genome browser). The location of rs6507 is highlighted with the red dotted line.

See the findings for rs6507 (Figure 6) as an example. In total, we identified 337 DEGs for rs6507. This rSNP resides in the coding sequence of the *CDC34* (ubiquitin-conjugating enzyme E2 R1) gene, which is required for the ubiquitin-mediated degradation of cell cycle G1 regulators and DNA replication initiation. The potential outcome given in the KEGG functional categories was linked to (i) metabolism (biosynthesis of antibiotics, pyrimidine, carbon metabolism, propanoate metabolism, and citrate cycle); (ii) GABAergic synapse, which mediates the majority of synaptic inhibition in the central nervous system; (iii) p53 signaling pathway, involved in multiple biological processes, including DNA damage repair, cell cycle arrest, apoptosis, and senescence; and (iv) the broadest category associated with these DEGs, namely, the pathways in cancer. In line with this, the root node for the PPI subnetwork is CCNE1, cyclin E1, an increased expression of which is a well-known tumorigenic factor and a prognostic biomarker in malignancies. Note that CDC34-mediated ubiquitination has been shown to enhance the proliferation capabilities of gastric cancer cells through an increased expression of cyclin E1 [48], that is, the target gene that we determined for this rSNP and the gene for which the largest network was constructed are functionally linked.

The additional description given in DO enrichment terms (Table S4) was an abnormal muscle function (‘muscular dystrophy’), difficulty in controlling eye movements (‘ocular motility disease’), and ‘congenital nystagmus’, which may either be a separate abnormality or associated with different underlying visual sensory and systemic disorder [49]. We have found that rs6507 strongly alters the binding motifs of two TFs, CTCF and ZBTB7B, and, therefore, may alter TF binding. Moreover, rs6507 falls into the ChIP-seq peaks of TF CTCF (Figure 6d), a multifunctional protein also shown to be involved in the development of malignancies [50].

**Figure 7:**
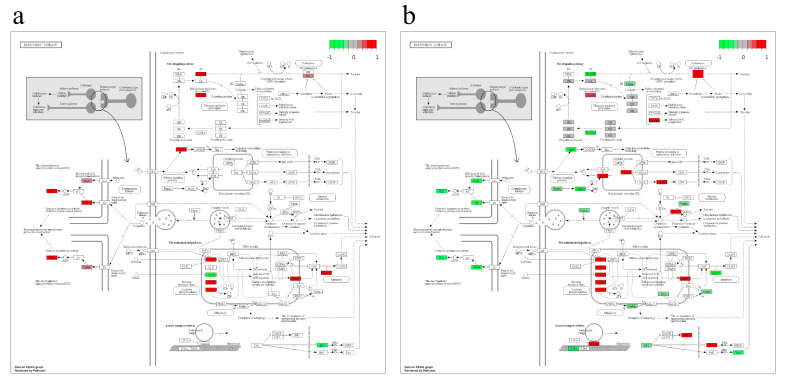
Graphical visualization of the KEGG pathway DEGs (Parkinson’s disease) was found to be associated with (**a**) rs16910241 and (**b**) rs56119169 variants in this study.KEGG pathway diagrams show the genes grouped in different functional units distributed along the pathway. Gene node colors show the direction of the change in expression.

Two other examples of visualization of the functional annotations for rs16910241 and rs56119169 polymorphisms are shown in Figure 7; we have found associations with Parkinson’s disease for both variants. Of special interest is the result for presynaptic terminals (Figure 7a,b; left panels). The proteins highlighted for the genes of the KEGG pathway acting at this stage for both rSNPs were guanine nucleotide-binding protein G(olf) subunit alpha (Gnal, highlight Golf), GNAI1(G protein subunit alpha i1), and PKA (cAMP-dependent protein kinase). However, these two rSNPs change the expression of these genes in different directions; namely, rs16910241 elevates the expression of all three genes, whereas rs56119169 decreases it. In addition, a set of DEGs with differently directed changes in their expression caused by these two rSNPs has also been observed for the mitochondrial stage, although the changes are somewhat less pronounced. Interestingly, rs16910241 and rs56119169 have different effects on p53 expression, which plays a fundamental role in the pathogenesis of neurodegenerative diseases [51]. Thus, our results suggest that many rSNPs from our list may be related to a number of potential phenotypic outcomes.

## 3. Discussion

A key problem in modern human genetics is to identify the DNA sequence variants (mainly SNPs) that influence different biomedical traits and to understand how genetic variation leads to phenotypic differences and complex diseases. It is currently evident that whole-genomeassociation study gives insufficient information for an adequate assessment of the effect of genetic variants on phenotype. Unlike GWAS, the recently developed approaches of functional genomics are initially focused on the detection of the SNPs associated with a change in the gene expression level. These approaches are mainly represented by eQTL analysis [32] and identification of allele-specific events [26,29,33,52], as well as some other genome-wide methods, such as massively parallel reporter assay (MPRA) [53]. An evident advantage of the analysis of allele-asymmetric events is that they make it possible to use relatively small samples (and even a single individual) for assessing the functional potential of most of the regulatory polymorphisms [54].

In this study, we sought to identify the functional variants that could affect gene expression (rSNPs) using an integrated analysis of allele-specific events in ChIP-seq and RNA-seq human datasets. To explore the functional relevance of rSNPs and their target genes, we used GWAS, GTExeQTL information, and network enrichment analysis.

In our earlier work, as in the majority of similar studies, we used the data obtained for cell lines [33]. However, the use of such material raises the question ofthe accuracy of rSNP identification. As is mentioned above, most cell lines are of a cancer origin with a characteristic high level of genome instability, which considerably complicates the identification of allele-asymmetric events [55]. Moreover, an important shortcoming when cancer cell lines are used is that an identified rSNP displays its functionality under conditions very far from the body’s natural context. The use of biopsy and/or surgery specimens of normal tissues resolves these problems. In line with this, the number of identified rSNPs in our study is significantly larger, amounting to 14,266 versus 1622 rSNPs found in our previous study on cell lines. However, the fact that we analyzed the whole genome rather than the known regulatory regions has most likely also contributed to the increase in the number of found rSNPs.

One of the most interesting findings in this study is that the degree of asymmetry of both ASB and ASE influences the effectiveness of rSNP search. This result suggests that a comprehensive analysis of these two types of allele-asymmetric events makes it possible not only to associate a genetic variant with the expression of certain target gene(s) but also to increase the prediction accuracy of its functionality. However, it is rather difficult to find some kind of phenotypic outcomes when using most of the functional approaches, including ourmethod. GWAS data are widely used in order to find a significant and statistically approved association of polymorphism with a phenotypic trait. In this work, we succeeded in linking 800 rSNPs to the GWAS-derived traits, including the rSNPs with strong LD according to NHD. The results are summarized in Table S2 and cover a number of phenotypes, including diseases. Their number is not too big but note that the rate of potentially functional variants annotated using GWAS data in other studies [28,29] usually does not exceed 5% of identified variants.

In addition, the difficulty in mapping SNPs to the genes with altered expression hinders the use of GWAS associations to clarify the molecular mechanisms underlying the corresponding trait [56]. Commonly, eQTL analysis is used for this purpose. Although this technique fails to reveal a direct association of an SNP with a phenotypic trait, it gives information about the genes for which the expression levels correlate with genetic variants. In theory, this makes it possible to reach a higher level of phenotypic outcome for at least part of rSNPs by revealing the functional links between these genes and reconstructing gene networks [57,58,59].

According to our data, 26% of the resulting rSNPswererepresented by the variants affecting gene expression from the GTExeQTL collection. The enrichment with GWAS-derived variants was considerably lower as compared with eQTLs when using any threshold: *p*-value cutoffs, predicted probabilities, or *z*-scores. This was expectable since both our approach and GTEx are aimed at the search for the correlation between a genetic variant and a change in gene expression.

Unfortunately, the use of GTEx for associating the found rSNPs to gene expression resulted in a few findings: we have found one to five eQTLs per one rSNP. Presumably, this is associated with the constraints used by the authors when forming the collection [60]. In addition, the GTEx Consortium analyzes only the genetic variants within 1 Mb of the target gene transcription start site to report an eQTL effect. Another factor is that very strict reliability thresholds were used when forming the collection. We were free from these constraints.

In addition, although GTEx is the largest eQTL database and currently may be the most actively used resource, not all eQTLs are included in GTEx. For example, Stolze et al. [61] identified thousands of eQTLs unique to endothelial cells but skipped by GTEx Project. Note that they used biopsy specimens rather than postmortem samples, as in the GTEx collection. We used the data for biopsy material and similarly succeeded in finding a considerable number of new eQTLs. We have not observed any significant prevalence of the rSNPs that overlap with eQTLs in any tissue from the GTEx collection; this may result from both the restrictions of the collection and the fact that the tissue-specific effect is not always considerably pronounced.

We searched for additional eQTLs using a representative independent RNA-seq brain dataset [44]. Our analysis of these RNA-seq data allowed us to detect much more links of rSNPs with genes up to 1000 eQTL effects per position. As an example of predicting the associated phenotypes, we considered three rSNPs from the set: rs6507 (C>T), a coding sequence variant within the *CDC34* transcript; rs16910241 (C>A), a coding sequence missense variant in the gene *H2AJ*; and rs56119169 (C>A), a variant residing in the regulatory region of the *MYL6* gene. The found eQTLs allow these rSNPs to be regarded as associated with neurological phenotypes. Presumably, this result reflects the fact that the RNA-seq data obtained for the brain were used as the initial data. Besides neurological phenotypes, other KEGG categories associated with the found polymorphisms, such as metabolic traits (Table S3), most likely reflect the best coverage of certain biological processes and the corresponding high abundance of these data in databases. Note that when conducting your own study, you can lower the thresholds and try to discover the way from an rSNP to a molecular phenotype with a certain increase in the overprediction error.

Thus, analyzing the public ChIP-seq and RNA-seq data, we have formed a new collection of rSNPs and functionally interpreted them. The phenotypic outcomes potentially associated with the found rSNPs were determined using the GWAS Catalog, analysis of the GTExeQTLs associated with these rSNPs, and functional analysis of the genes differentially expressed in the brain of the individuals with the genotypes differing in these rSNP alleles.

## 4. Materials and Methods

### 4.1. Human NGS Data

No experiments involving human subjects conducted by the authors are described in this paper. All used human datasets from the open access are under ethical consent agreements as stated in authorized submissions.

ChIP-seq profiling of the active promoter mark H3K4me3 obtained for PAECs and transcriptome sequencing data for the same samples were available at NCBI under accession number GSE126325. We used only the data for controls (donor lungs, *n* = 19) [43]. A total of 9.4 Gb of data was obtained, which was, on average, 170 Mb of data per each ChIP-seq library and 320 Kb per each RNA-seq library.

Transcriptome sequencing of 281 clinically annotated human postmortem brain tissues (anterior cingulate gyrus, dorsolateral prefrontal cortex, and nucleus accumbens) for five conditions (schizophrenia, bipolar disorder, major depressive disorder, autism, and controls) [44] wasavailable at NCBI under accession number SRP073813. In total, 14.3 Mb of data were obtained; on average, 48.2 million reads per library.

Genome sequencing data of 2504 individual genomes sampled from five super populations (AFR, EAS, SAS, AMR, and EUR) were available from the 1000 Genomes Project release Phase 3 [62].

The SNP calls were downloaded in VCF format from the release directory on the EBI FTP site (http://ftp.1000genomes.ebi.ac.uk/vol1/ftp/data_collections/, accessed on 29 April 2020).

### 4.2. Open Access Resources

The categories of gene elements, such as promoters, untranslated regions (UTRs), and transcription start sites (TSSs), for autosomal human genes were obtained from GENCODE release 37 [63].

The list of consensus human enhancers was obtained from the EnhancerAtlas 2.0 database [47]; all items were experimentally validated by a dozen of high-throughput technologies. The enhancer–target gene interactions were predicted using the EnhancerAtlas 2.0 computational tools.

The SNP-trait associations were obtained from the GWAS Catalog [3] with the summary statistics of 248,356 associations as of January 2021. The associations were not segregated by trait.

The eQTLs for human postmortem tissues were obtained from the GTEx portal on 11/19/20 and dbGaP accession number phs000424.v8.p2. The significance level threshold for eQTL effects was reported in [64].

### 4.3. NGS Data Preprocessing

#### 4.3.1. Quality Filtering

The raw reads from ChIP-seq and RNA-seq experiments were trimmed for quality (phred ≥ 20) and length (bp ≥ 32) using Trimmomatic v. 3.2.2 [65]. Illumina adapters were cut off.

#### 4.3.2. Genomic Alignment and SNP Calling

Then, Bowtie2 was used to align the reads to the GRCh38 human reference assembly [66]. PCR duplicates were discarded with Picard tools to reduce their effect on the accuracy of subsequent variant calls. The threshold of QMAP of at least 25 by SAMtools [67] was set for mapping quality.

After the first alignment step, the high-quality sequencing reads that passed thresholds were assembled for further SNP discovery pipeline using SAMtools pileup, R tools (https://www.r-project.org/, accessed on 2 April 2021), and Perl scripts. The sex chromosomes and mitochondrial DNA were not analyzed.The SNPs located 5 bp from both ends of short insertion–deletion variants were also removed. For a specific set of variant calls (in VCF format), the GATK FastaAlternateReferenceMaker was used to replace the reference bases in polymorphic positions with the bases representing the alternative alleles discovered in each sample from PAECs and brain datasets. The final alternative reference sequence was separately reported for each ChIP-seq or RNA-seq sample. Then the read alignment was improved through the second alignment step to both GRCh38 and appropriate alternative references. Locations of all heterozygous SNPs were converted to a standardized format that described the alleles. The position was considered heterozygous when the minor allele coverage was no less than 10.

### 4.4. Assessing Allele-Specific Binding and Expression Events

A bias analysis was performed as earlier described [33]. Briefly, after the re-alignment step, we counted the number of reads covering each allele at heterozygous positions (SNPs with ASB in ChIP-seq data and heterozygous SNPs within targeted genes in RNA-seq data) using both custom Perl scripts and SAMtools. The ASB and ASE events were determined as the share of reads with the reference allele based on statistically significant enrichments (odds ratio ≥ 1.5) or depletions (odds ratio < 1.5), respectively (padj ≤ 0.1). The significance of the bias was determined according to a binomial model implemented in R with the null hypothesis that both alleles of a heterozygous SNP would be equally covered and represented in the data. The degree of asymmetry for allele-asymmetric events, such as ASB and ASE, was determined by the parameter |log_2_FC|, where FC is a fold change.

### 4.5. Z-Test

Different approaches are used to combine the results of hypothesis testing from several independent experiments. We used a weighted *z*-test [45] to calculate the combined *p*-value of two binomial tests by ChIP-Seq and RNA-Seq experiments.

### 4.6. Evaluation of Linked SNP Pairs by HAMMING Distance

According to the concept of Hamming distance from coding theory [68], defined as the number of different bits at the same positions of two linear datasets, we assumed that if two alleles of two different SNP positions occurred in the same haplotype combination in the population than these two SNPs should be closely linked. The normalized Hamming distance (NHD) was used to measure LD and was calculated as in [69]:

$$NHD\;\left(cSNP,\;rSNP\right)=\frac{\sum_{i=1}^{n}\left|\mathrm{Ri},\;\mathrm{rSNP}-\mathrm{Ri},\;\mathrm{cSNP}\right|}{\sum_{i=1}^{n}\left|\mathrm{Ri},\;\mathrm{rSNP}+\mathrm{Ri},\;\mathrm{cSNP}\right|}$$

where *R_ij_* = 0, 1, or 2 is a number of rare alleles in the *j*th position of the *i*th person from the 1000 Genomes dataset (*n* = 2504). The Hamming distance measurement was normalized to the number of rare alleles found in the 1000 Genomes dataset for each position. The less the Hamming distance, the more strong relationship is observed between two SNP markers. The minimum of NHD is zero if the rare alleles in one position correspond in all cases to rare alleles in another position; otherwise, NHD amounts to unity if one SNP from the pair is associated with the major allele and the other, with the rare allele for all individuals.

Estimation of conditional probability is another way to predict an rSNP based on the existence of a linked coding SNP in RNA-seq.

Assume that allele “A” for the rs738904 genotype is obtained in RNA-seq (Table 3). What is the probability that the same person has the “G” allele of this polymorphism in at least one copy of chromosome 22? For the rs738904, let us denote as “A*” the genotype of an individual who carries one “A” allele, while the other allele can be (in this case) “A” or “G”. In an analogous manner, for the rs7289432, let us denote as “G*” the genotype of an individual who carries one “G” allele, while the other allele can be (in this case) “A” or “G”. Then, we can use the conditional probability equation to find P{“G*”|“A*”} = P{“G*”,“A*”}/P{A*}.

The probability estimates werecalculatedfor the sample of 2504 individuals as:

P{“G*”,“A*”} = (1042 + 5 + 7 + 352)/2504 = 1406/2504 and

P{“A*”} = (1054 + 357)/2504 = 1411/2504.

Then, P{“G*”|“A*”} = 1406/1411 = 0.996.

Thus, if the “A” allele is obtained in RNA-seq for the rs738904 polymorphism, the “G” allele for the rs7289432 polymorphism will be present at a high probability.

When predicting the presence of regulatory polymorphisms, we used a conditional probability of at least 0.9 and selected the polymorphisms with the least NHD from all predicted regulatory polymorphisms.

### 4.7. Transcription Factor Motif Disruption Analysis

We used motifbreakR package [70] to predict the effect of rSNPs on TF motifs. Position-specific weight matrices of the candidate TFs retrieved from the HOCOMOCO database [71] were used to generate the motif consensus sequences.

The list of rSNPs that strongly altered the motifs was then overlapped with the Transcription Factor ChIP-seq Peaks track, a comprehensive set of 340 human factors in 129 cell types from ENCODE 3 [72] using R. We additionally selected a set of rSNPs breaking TF motifs and falling to the ChIP-seq peaks for the same TFs according to ENCODE data.

### 4.8. Differential Expression Analysis

We used the DeSeq2 R package [73] to identify the genes differentially expressed between the groups of people of different genotypes (at least three individuals in the group for each genotype). RNA-Seq data for three brain structures for each individual were analyzed separately [44].

### 4.9. Construction of Protein–Protein Interaction Networks and Functional Annotation

We used STRINGdb R package [74] as an interface to the STRING database (https://www.string-db.org, accessed on 2 April 2021) to construct PPI networks for the rSNPs associated with eQTLs, for each separately, and to perform further functional annotation.

We used theclusterProfiler R package [75] for additional annotation of the DEGs when analyzing the brain RNA-seq dataset. The DEGs for each analyzed rSNP position were tested independently for representation in Gene Ontology (GO) Biological Process terms [76], Disease Ontology (DO) terms [77], and associated Kyoto Encyclopedia of Genes and Genomes (KEGG) pathways [78].

### 4.10. Statistical Analysis

The statistical analysis was performed using R software. Correlation rank-based metrics were applied to the calculated *z*-scores and predicted probabilities under the null hypothesis of a positive correlation between the means of the integral parameter and the incidence of rSNP to be a GTEx eQTL or a GWAS trait-associated SNP. The outcome results were interpreted according to the degree of association as strong (*r* = 0.7–1), moderate (*r* = 0.5–0.7), or low (*r* = −0.5) after taking into consideration the significant correlation values. The data on logistic regression were subject to one-way ANOVA using R to analyze the parameters that influence the probability of the rSNPs entering GTEx eQTLs or GWAS. The predictions of the logistic regression models calculated taking into account two or three parameters were set as independent variables. Benjamini–Hochberg [79] false discovery rate adjustments were applied to significant values to correct for multiple testing with a threshold of padj<0.1 using p.adjust function in R (unless otherwise stated).

## Figures and Tables

**Table 1:** Logistic regression analysis prediction parameters. |log_2_FC1| is the proportion between the coverage of two alleles for the SNP with an ASB effect; |log_2_FC2|, the same for the position with an ASE effect; |log_2_FC1/FC2| reflects the difference in asymmetry for SNP in independent ChIP-seq and RNA-seq experiments; Sign:significance (* *p*-value < 0.05; *** *p*-value < 0.001), dispersion parameter for binomial family was taken to be 1.

Parameter	Regression Coefficient	Std. Error	*p*-Value	Sign
|log_2_FC1|	−0.547335	0.009193	<2 × 10^−16^	***
|log_2_FC2|	−0.022103	0.008592	0.0101	*
|log_2_FC1/FC2|	−0.125600	0.010718	<2 × 10^−16^	***

**Table 2:** Gradual enrichment of SNP sets with GTExeQTLs and GWAS associations. ASB, allele-specific binding bias; ASE:allele-specific expression bias; eQTLs:GTEx expression quantitative trait loci; GWAS:all available GWAS-derived SNP-trait associations; *n*: the number of SNPs; %: percentage relative to the total number of SNPs; pp:predicted probabilities.

Identified SNPs	*n*	Overlapping with All GTEX eQTLs, %	Overlapping withthe GTExeQTLs with *p*-Value < 0.1, %	Positions Contained in GWAS Catalog, %
Heterozygous SNPs	~4.3 *×* 10^6^	13	8	2.1
SNPs with ASB (*p*-value < 0.1)	58,191	15	10	2.5
SNPs with ASE (*p*-value < 0.1)	230,553	15	10	2.7
SNPs with both ASB and ASE (both *p*-values < 0.1)	20,321	23	18	3.0
SNPs with both ASB and ASE (*z*-test *p*-values < 0.0005)	14,898	23	18	3.1
SNPs selected by predicted probabilities, pp > 0.1929408	14,543	26	20	3.5
SNPs selected by log regression and *z*-score	10,318	26	21	3.7

**Table 3:** Contingency table of the rs7289432 and rs738904genotypes for a sample of 2504 individuals from the 1000 Genomes dataset.

rs7289432 22chr:19171209	cSNP (rs738904) 22chr:19179872			Total Number of Genotypes in 1000 Genomes
	CC	AC	AA	
AA	1084	5	0	1089
AG	9	1042	5	1056
GG	0	7	352	359
Total number of genotypes in 1000 Genomes	1093	1054	357	2504

## Data Availability

Publicly available datasets were analyzed in this study. This data can be found at NCBI (GSE126325 andSRP073813) and from the 1000 Genomes Project release Phase 3 (http://ftp.1000genomes.ebi.ac.uk/vol1/ftp/data_collections/, accessed on 29 April 2020). The data presented in this study are available in Supplementary Material. The data on all regulatory SNPs found in this study are available on request from the corresponding author. These data are not publicly available due to their large amount.

## Supplementary Materials

The following are available online at https://www.mdpi.com/article/10.3390/ijms22147240/s1.
